# Beri-Beri and Wernicke Encephalopathy in a Thirty-Year-Old Male

**DOI:** 10.7759/cureus.24692

**Published:** 2022-05-03

**Authors:** Madalena Lobao, Maria Beatriz Sampaio, Miguel Sousa Leite, Felisbela Gomes, Joao M Silva

**Affiliations:** 1 Internal Medicine, Hospital de Santo Antonio dos Capuchos, Centro Hospitalar Universitario de Lisboa Central (CHULC), Lisbon, PRT; 2 Internal Medicine, Hospital de Santo Antionio dos Capuchos, Centro Hospitalar Universitario de Lisboa Central (CHULC), Lisbon, PRT; 3 Internal Medicine, Centro Hospitalar Universitário de Lisboa Central (CHULC), Lisbon, PRT

**Keywords:** reversible cardiomyopathy, thiamine deficiency, bariatric surgery, wernicke encephalopathy, beri-beri

## Abstract

A 30-year-old Turkish male was found lethargic and surrounded by vomit. At the hospital, severe hypernatremic dehydration and acute kidney failure were evident. His conscious level improved with fluid resuscitation. A differential diagnosis of altered mental status was considered. A complete clinical triad of Wernicke encephalopathy (WE), supported by MRI findings, was compatible with thiamine deficiency. Previous bariatric surgery was later confirmed. Despite no clinical signs of heart failure, a high level of NT-proBNP (N-terminal prohormone brain natriuretic peptide) and a dilated, hypokinetic myocardiopathy detected on the echocardiogram led us to assume beri-beri heart disease. High-dose intravenous thiamine, ACE (angiotensin conversing enzyme) inhibitors, beta-blockers, and physical therapy were initiated with remarkable improvement in his clinical condition.

## Introduction

Vitamin deficiency due to malnutrition is particularly common in economically disadvantaged locations. Attempts to deal with obesity in high-resource settings, such as restrictive bariatric surgery, are becoming a cause of acute vitamin deficiency syndromes such as thiamine deficiency, but also delayed copper and B12 vitamins. Thiamine is a co-enzyme required for glucose generation and neurotransmitter production. Storage exhaustion can start as early as two to three weeks [1]. Thiamine deficiency leads to neuronal loss, neuromuscular dysfunction, and oxidative stress, which is reflected in muscle weakness, areflexia, and progressive sensorimotor neuropathy (dry beri-beri).

Cardiovascular manifestations happen in the absence of thiamine since cellular metabolism is compromised. An accumulation of adenosine triphosphate leads to a reduction in systemic vascular resistance via direct vasomotor depression and a compensatory high-output state with increased blood volume (wet beri-beri) takes place. Peripheral vasodilation, arteriovenous shunting, activation of the renin-angiotensin-aldosterone system, dyspnea, edema, and dysrhythmias are often present [2].

Wernicke encephalopathy (WE) is characterized by a triad of encephalopathy, nystagmus, and ataxia. Damage in regions of high demand for thiamine (thalamus, mammillary bodies, basal forebrain, and cerebellum) results in anterograde and retrograde amnesia, confabulation, cognitive impairment, and memory deficits (Korsakoff syndrome, KS). Untreated, the estimated mortality of WE is 90%, and among recognized cases, it is as high as 25%. Up to 80% of patients who survive develop KS [3].

## Case presentation

A 32-year-old Turkish male living in Portugal for six months was brought to the emergency department, lethargic and covered in vomit. He had received Roux-en-Y bariatric surgery 10 months prior to the admission. Such information was not available until a few days later when contact with the family was possible. Nutritional supplements had been prescribed, but he failed to take them. His family was concerned that he had become increasingly isolated, with memory impairment and personality changes. There was no history of drug abuse, although he would occasionally consume alcohol and cigarettes.

Upon admission, he was extremely dehydrated, tachycardic (155 bpm) but normotensive, with 3-4 mm hyporeactive pupils, Glasgow scale 8, apyretic, without focal neurological or meningeal signs. The glucose level was normal. Naloxone and flumazenil failed to improve his condition. Laboratory analysis suggested hypernatremic hypernatremia with acute kidney injury and lactic acidosis (sodium 153 mmol/L, potassium 3.6 mmol/L, chloride 121 mmol/L, urea 124 mg/dL, creatinine 1.74 mg/dL, lactate 7.1 mmol/L, pH 7.23, and HCO3- 17.5 mmol/L). Ethanol and drug screens were negative.

After fluid resuscitation, his consciousness level improved; inattention, short-term memory loss, predominantly horizontal nystagmus (bilateral gaze-evoked, short period of upbeat nystagmus) with VI cranial nerve palsy, severe arm (link arm slightly worse) and limb ataxia were evident in his neurological evaluation. A mild burning sensation on the limbs and diminished deep tendon reflexes (biceps, brachioradialis, triceps, patellar, achilles) were also noted. The plantar response was normal. Proprioception was preserved. No stigmata of liver disease were evident; abdominal striae and skin excess were, however, noticed.

**Figure 1:**
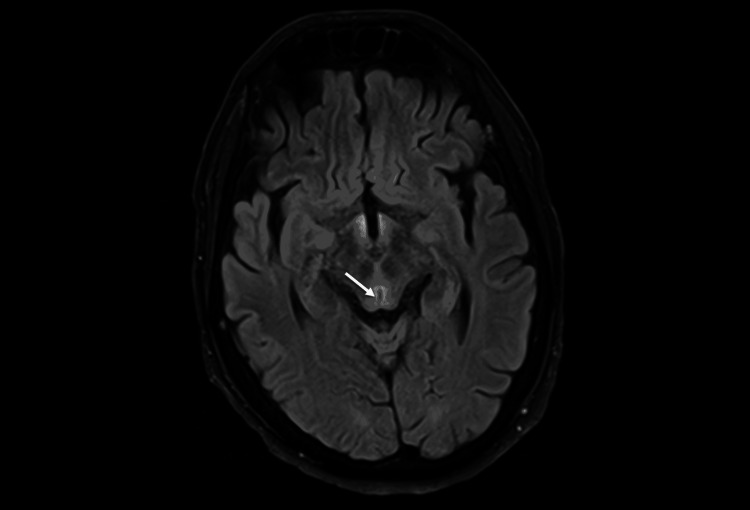
Axial T2 FLAIR, periaquedutal grey matter hyperintensity FLAIR: fluid-attenuated inversion recovery

**Figure 2:**
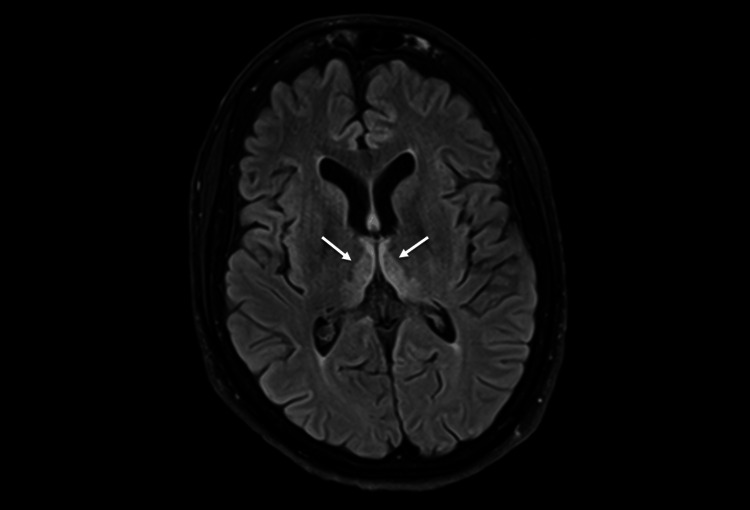
Axial T2 FLAIR, thalamic dorsomedial bilateral symmetric hyperintensity FLAIR: fluid-attenuated inversion recovery

MRI findings were compatible with Wernicke encephalopathy (symmetrical increased signal involving the dorso-medial thalami, tectal plate, periaqueductal and mammillary bodies; Figures 1-2).

Two lumbar punctures were performed. Cerebrospinal fluid analysis was innocent (no pleocytosis, glucose, cell count normal) and infectious causes were ruled out (PCR/culture for viral, fungal, and bacterial organisms). Electroencephalograms did not suggest other etiologies of encephalopathy.

His first electrocardiogram (EKG) showed T-wave inversion in the precordial leads without troponin elevation. Chest pain and clinical findings of heart failure were absent. A transthoracic echocardiogram showed a dilated left ventricle, global hypokinetic myocardium, and an ejection fraction of 40%. NT-proBNP (N-terminal prohormone brain natriuretic peptide) measurement was elevated (1270 pg/mL, normal <450 pg/mL). No ischemic lesions were found on cardiac angio-computerized tomography (CT).

High-dose thiamine supplementation was started (500 mg iv tid for five days) [4]. The patient was admitted to the intermediate care unit for neurological status surveillance and further diagnostic evaluation. His kidney function normalized within the first few days and sodium levels reached their normal range at an appropriate rate. Nutritional support was optimized and other vitamins were supplemented: B12 (371 pg/mL), iron (iron 36 µg/dL, total iron-binding capacity 107 µg/dL, transferrin saturation 31%), folic acid (2.1 ng/mL), vitamin D (10 ng/mL), and calcium (corrected to albumin 8.7 mg/dL) [5]. Disease-modifying therapy for heart failure was discussed with cardiology, and angiotensin conversing enzyme (ACE) inhibitors and beta-blockers were introduced. Physical therapy and cognitive training were initiated.

EKG changes normalized within a few days of treatment. A second echocardiogram performed a month later showed significant recovery of ejection fraction (48%). NT-proBNP levels decreased to 125 pg/mL. A second brain MRI also marked improvement. Upon discharge, the patient was able to walk even though some degree of ataxia was present.

A neuropsychological assessment was advised since it ought to be performed in the patient’s native language. Further follow-up by neurology, cardiology, physical therapy, and cognitive training was also recommended. Supplementation with complex B vitamins, iron, copper, zinc, calcium, vitamin D, and oral thiamine supplement (benfotiamine 150 mg bid) for four to six weeks was suggested after discharge. Nutritional re-evaluation within four weeks was proposed. The patient has recently emailed us to report that he is being checked regularly, has gained about 10 kg, is taking his supplements, and continues to improve.

## Discussion

A young adult was brought to the emergency department with an altered mental status. A potentially fatal condition underlying an altered mental state has a wide range of differential diagnoses. Our patient had a history of bariatric surgery. Early diagnosis was challenging, but his neurological symptoms were compatible with non-alcoholic Wernicke encephalopathy. This case also illustrates other consequences of thiamine deficiency: peripheric neuropathy (dry beri-beri) and subclinical cardiac dysfunction (wet beri-beri). Anamnesis, neurological exam, laboratory workup, and radiologic evaluation excluded other causes of central nervous system dysfunction. A favorable response to treatment corroborated our findings and prevented more devastating consequences.

The first obstacles we encountered in our diagnostic approach were the language barrier and the absence of relatives/close friends who could provide accurate background information.

Alcohol and drug intoxication were highly probable but rapidly ruled out. Skin excess, abdominal striae, and scars were clues to suspecting previous bariatric surgery. Thiamine deficiency was strongly considered once other neurological signs were recognized: abnormal eye movement (nystagmus, VI nerve palsy), ataxia, and a sudden altered mental state. MRI findings were typical of WE.

Thiamine in whole blood testing is not available in our laboratory. Nevertheless, thiamine levels are not required for definitive diagnosis and should not delay the prompt institution of supplementation [6].

The neuropsychological assessment establishes the diagnosis and progression of Korsakov syndrome [7]. Adequate vitamin supplementation and neurocognitive rehabilitation are essential for a successful outcome.

If disease-modifying therapy for heart failure plays any role, it is not clear to us yet. Guidelines from the European Society of Cardiology include thiamine deficiency as a cause of cardiomyopathy, but no specific evaluation is emphasized. Until other causes of cardiac failure were excluded, we have opted for all the interventions that could possibly be beneficial [8]. Since cholesterol levels were normal, statins were not initiated (the risk of poly-neuropathy outweighed the benefit) [9].

## Conclusions

The importance of therapeutical adherence and proper follow-up after restrictive gastric surgery cannot be overemphasized, and the consequences of thiamine deficiency must be explained to patients and families. In this particular case, moving to another country during pandemic times was one of the reasons contributing to the distance our patients from the health system.

Prompt recognition and correction of nutritional deficiencies according to a specific geographical and economical setting are crucial. Acute vitamin deficiency syndromes such as thiamine deficiency should be suspected in cases of altered status and bariatric surgery questioned as a prevalent cause in high-income settings. Clinicians should be aware, as outlined by this case, that there can be life-threatening with potentially disabling consequences from inadequate addressing of neurological nutritional deficiencies syndromes.
